# Longitudinal Study of Body Composition and Energy Expenditure in Overweight or Obese Young Adults

**DOI:** 10.1038/s41598-020-62249-8

**Published:** 2020-03-24

**Authors:** José Carlos Fernández-García, Ismael Gálvez-Fernández, Pere Mercadé-Melé, Juan Gavala-González

**Affiliations:** 1 Department of Didactics of Languages, Arts and Sport, University of Malaga, Andalucía-Tech, Malaga, Spain, IBIMA, Malaga, Spain; 2 Department of Statistics and Econometrics, University of Malaga, Andalucía-Tech, Malaga, Spain; 3 Department of Physical Education and Sports, University of Seville, Seville, Spain

**Keywords:** Lifestyle modification, Weight management

## Abstract

The aim of this study was to compare the effects of an aerobic training program with a strength training program on body composition and energy expenditure in overweight or obese (29.06 ± 3.49 kg/m^2^) young adults (21.96 ± 1.90 years). Subjects (N = 109) were randomly assigned to one of three groups: a control group (CG), an aerobic training (AT) group and a strength training (ST) group. Training took place over twelve weeks comprising three sessions per week with each session lasting 60 to 90 minutes. Before and after the program, weight, height, body mass index, lean mass percentage and fat mass percentage were evaluated. In addition, The International Physical Activity Questionnaire-Short Form (IPAQ-SF) was used to estimate energy expenditure. The results of both aerobic training and strength training produced statistically significant improvements in weight (AT-CG = −2.892 kg; ST-CG = −2.986 kg); BMI (AT-CG = −1.075 kg/m^2^; ST-CG = −1.118 kg/m^2^); total body fat (AT-CG = −1529.172 g; ST-CG = −763.815); and total body fat percentage (AT-CG = −1.421%; AT-ST = −0.855%). These two exercise prescription models were therefore useful in reducing overweight and obesity, which could have an impact on improving the health and quality of life of individuals with these characteristics.

## Introduction

In the current population, a number of non-communicable diseases are strongly linked to overweight and obesity and affect a wide range of people in all age groups, including young adults^1^. A determining factor in the increased weight of present-day society is sedentary behavior, i.e. physical inactivity occupies a considerable part of leisure time, which, as indicated by Salonen *et al*. (2015) and Silva *et al*. (2019), leads to a low level of METs (Metabolic Equivalent of Task), and a worsening of body composition, which is directly related to an increased likelihood of different diseases^1–3^. In fact, physical inactivity is identified by the WHO as the fourth leading risk factor for mortality worldwide^4^.

Overweight and obesity have become a serious global public health problem, to the extent that currently more than 38% (39% of men and 40% of women) of the adult population is overweight or obese^5^, and the trend is for this percentage to increase in the coming years^6,7^. Although weight gain occurs at all ages in both developed and developing countries, it is most rapid in young adults^7^. This increased weight, carrying excess weight, and obesity are associated with poorer quality of life^8^ and with different diseases^5,7^, whereas weight loss due to physical activity leads to cognitive^9^ and metabolic^10–12^ improvements.

Different methods have been used to treat overweight and obesity in adults^13–15^ these have included training and diet programs^15–17^, diet programs alone^18–20^, and training exclusively, the latter being the focus of our study. Among training programs, those based on aerobic training (AT)^21–32^ and strength training (ST)^33–44^ are of particular note. The human body is a highly dynamic and plastic system in terms of adaptation responses to exercise since, according to the type of exercise and strain on muscle fibers, an improvement in functional, structural or metabolic properties is demonstrated^45^. Aerobic endurance and strength training stimuli represent two important extremes^46^.

Good results have been obtained with AT largely due to improved mitochondrial function and the oxidation of fatty acids^19,20^. The results of AT on anthropometric variables are contradictory, since improvements occur in some studies^21–24^ but not in others^26,27,29^. It should also be noted that in several studies it is not possible to determine whether the improvement is produced by physical activity, diet or a combination of both^25,28–30^. The majority of the studies on ST show improvements in anthropometric parameters^36–44^. However, similar to the studies on AT, some do not determine whether this improvement is due to diet or training^36,37^. There are also studies in which no improvements are seen^34,35^. The improvements found lead to better health^40–44^.

In recent years, physical activity has become a pillar for public health strategies and programs due to the many benefits as well as the consequences of physical inactivity, which is considered the fourth risk factor for global mortality^47^. The International Physical Activity Questionnaire-Short Form (IPAQ-SF) has been used to determine the level of physical activity carried out by individuals. This questionnaire is supported by the WHO, is validated at the international level^48^, and has been used in various intervention studies in adults with satisfactory results^49–51^.

The information on weekly activity received through the questionnaire is recorded in METs per minute and week^52^. METs are a method of calculating energy requirements and are multiples of the resting metabolic rate. The unit used, the MET-minute, is calculated by multiplying the MET corresponding to the type of activity by the minutes the activity is performed in a day or in a week. In this study, results are expressed in MET-minutes per week^53^. Through this registry we obtain a series of values that allow us to estimate the quantity and quality of physical activity carried out and that, in our case, was controlled before and after the twelve-week training period, following the same model that has been used in other studies^54,55^.

Currently, few studies exist in which the participants were overweight or obese young adults who participated in a training program with no influence on their diet. These studies showed weight loss, reduced body mass index (BMI) and total body fat percentage after a 12-week AT program^21,22^. Several studies have been identified involving overweight and obese subjects who undertook AT programs who were not young adults, in which statistically significant improvements were detected in weight loss (−1.60 kg and −1.69 kg) and BMI (−1.36 kg/m^2^ and −2.03 kg/m^2^)^29,32^. Other studies that carried out AT programs in young people who were not classified as overweight or obese, and as in previous research in which AT was prescribed, reported weight loss (−4.3 kg and −2.1 kg)^25,31^ and an improvement in BMI (−1.3 kg/m^2^)^25^. In the literature, the same results have been found with ST. Some studies in overweight and obese subjects who participated in ST programs, although not in young adults, detected weight loss (−4 kg; −3 kg; −2.5 kg) and a decrease in BMI (−1.6 kg/m^2^; −1.2 kg/m^2^; −2.1 kg/m^2^)^37,38,40^. The studies by Gálvez (2017) and Ibrahim *et al*. (2018) carried out ST interventions in young people who were not overweight or obese, resulting in weight loss (−2.4 kg and −1.1 kg) and a slight improvement in BMI (−0.8 kg/m^2^ and −0,3 kg/m^2^)^41,44^.

The main innovation provided by our study is the treatment of overweight and obesity in young adults through the exclusive implementation of a physical exercise program, without diet modification. Accordingly, the main objective of this study was to compare the effects of an AT program and an ST program on body composition and energy expenditure in a target population of overweight or obese young adults aged 18–25 years.

## Methods

### Design and participants

To participate in the REPHASO (Rejecting Obesity and Overweight through Healthy Habits) research project, the subjects had to have a BMI ≥ 25 kg/m^2^, a figure that the WHO considers overweight^5^. The sample comprised 109 young adults between the ages of 18 and 25 years (21.96 ± 1.90 years) with a BMI = 29.06 ± 3.49 kg/m^2^, calculated from reported weight and height. Proportional allocation was performed for male and female participants in all groups.

Participants were recruited from a variety of sources and completed an online form in order to be initially selected based on their BMI. Once selected, they met with the project coordinator who explained the nature of the study, indicating that their anonymity would be preserved at all times, following the ethical considerations of Sport and Exercise Science Research^56^ and with the principles included in the Declaration of Helsinky^57^, which defines the ethical guidelines for research involving human subjects. In addition, the present study has been approved by the Ethical Committee of experimentation of the University of Malaga (CEUMA). Written inform consent was taken from the participants. Similarly, throughout the intervention and thereafter, we acted in accordance with the Spanish regulations established in the Organic Law 15/1999, of 13 December, regarding the protection of personal data.

### Instruments

Weight was measured to the nearest 0.1 kg using a Tanita model BC730 scale. Height was measured to the nearest 0.1 cm with a SECA model 213 portable stadiometer. Body fat, lean mass, percentage of total fat and total body mass were assessed using a dual-energy x-ray densitometer (DXA, Hologic Explorer, United States) (DXA). In addition, the participants completed a 24-hour dietary recall^58,59^ and the IPAQ-SF. This questionnaire consists of seven questions which have acceptable measurement properties for monitoring physical activity levels for adults aged 18 to 65 in various settings and also reports the number of METs^55^. Several studies have validated the reliability of the IPAQ-SF to report the number of METs, as significant results were obtained compared to other tests such as accelerometry or podometry^52,60–62^.

### Procedure

Each participant, while barefoot and wearing light clothing, underwent an initial anthropometric evaluation of weight and height to calculate BMI. Subsequently, whole-body densitometry was performed. At the end of the evaluation, each participant completed the self-administered version of the IPAQ-SF questionnaire and a 24-hour dietary recall.

Following the initial evaluation, the subjects were randomly divided into three groups: The Control Group (CG), which did not carry out a physical activity program, and the experimental group, which in turn was divided into two groups. Both experimental groups were prescribed a program of 12 consecutive weeks. The AT group performed aerobic training (Table 1) and the ST group performed strength training (Table 2). For both programs, each week included three days of training lasting between 60–90 minutes per session. These sessions were supervised by a personal trainer who ensured attendance, the correct execution of tasks and the intensity of the sessions as well as excluding from the study those subjects who did not comply with at least 90% participation. The training period was divided into three stages for the AT and four for the ST, which increased in intensity. These stages were regulated according to the subjective perception of exertion of the participants, through the Börg scale^63^.

**Table 1:** Exercise prescription design for the AT group.

Stage	Content
1	Training for 60–90′ walking and cycling with no gradient. Börg scale 5–6.
2	Training for 60–90′ walking, running and cycling alternating phases with a slight gradient and no gradient. Börg scale 6–7.
3	Training for 60–90′ walking, running and cycling with most phases with a gradient. Börg scale 7–8.

**Table 2:** Exercise prescription design for the ST group.

Stage	Content
1	Exercises with a maximum of 10 repetitions, with a recovery of 10–15″ between series. Börg scale 5–6.
2	Exercises with a maximum of 12 repetitions, with a recovery of 10–15″ between series. The number of repetitions and the difficulty of the exercises increase slightly with respect to the previous stage. Börg scale 6–7.
3	Exercises with a maximum of 12 repetitions, with a recovery of 10–15″ between series. The difficulty of the exercises increases with respect to the previous stage. Börg scale 6–7.
4	Exercises with a maximum of 15 repetitions, with a recovery of 5–10″ between series. The difficulty of the exercises increases with respect to the previous stage and the recovery time decreases. Börg scale 7–8.

At the end of the twelve-week training period, the participants were re-evaluated using the same procedure used in the initial evaluation.

### Data analysis

The data were collected in an Excel spreadsheet, including age, height, weight, BMI, 24-hour dietary recall reponses, IPAQ-SF responses, and participant affiliations. Statistical analyses were performed using the Statistical Package for Social Sciences, version 20 (IBM Corp., NY, USA).

An analysis of covariance (ANCOVA) between the experimental groups was performed on several dependent variables: weight, BMI, total body fat, total lean mass, total body mass and total body fat percentage. The type of training was considered as an independent variable with three levels (CG and two experimental groups: AT and ST) and the respective pretest scores of each dependent variable as the covariate. Although randomization results in well-balanced treatment groups, covariate adjustment is desirable when pretest and posttest data are strongly correlated^64^. In this case, the use of pretest scores reduces the variance error and tests are more powerful^65^. With the aim of analyzing physical differences, the differences between the groups were estimated. Data were checked for normality using the Kolmogorov-Smirnov test. To analyze multiple comparisons between pairs of means of the dependent variables adjusted for the covariance, Bonferroni tests were carried out. Through this test, we evaluated the differences among multiple means. Finally, with the aim of analyzing the differences between the responses.

## Results

Table 3 shows the adjusted means, F statistics and p-values. The analysis shows significant differences between the different groups in the variables weight (F = 9.35; p = 0.000), BMI (F = 11.13; p = 0.000), total body fat (F = 3.228; p = 0.044) and total body fat percentage (F = 4.543; p = 0.013). For the variables weight and BMI posttest, the adjusted means were lower in the ST group; and for the variables total body fat and total body fat percentage, the adjusted means were lower in the AT group. No significant changes were observed in total lean mass and total body mass.

**Table 3:** Adjusted means for the control group, the aerobic group and the strength group for each dependent variable, F-statistic and p-value.

Variables	Control group	Aerobic group	Strength group	F	*p*
Weight (kg)	85.520	82.627	82.534	9.35	0.000***
BMI (kg/m^2^)	29.617	28.542	28.500	11.137	0.000***
Total body fat (g)	28497.048	26967.876	27733.234	3.228	0.044**
Total lean mass (g)	55179.274	55357.291	54841.72	1.179	0.312
Total body mass (g)	83668.268	82337.86	82567.789	1.421	0.246
Total body fat percentage (%)	34.356	32.935	33.79	4.543	0.013**

*p < 0.1; **p < 0.05; ***p < 0.01.

Table 4 analyzes multiple comparisons between pairs of means of the dependent variables adjusted for the covariance by the Bonferroni test.

**Table 4:** Statistical Analysis: Bonferroni Multiple Comparison Test.

Variable	Bonferroni Multiple Comparison Test	Mean Difference	*p*
Weight (kg)	Aerobic – Control	−2.892	0.001***
	Strength – Control	−2.986	0.000***
	Aerobic – Strength	0.093	1.000
BMI (kg/m^2^)	Aerobic – Control	−1.075	0.000***
	Strength – Control	−1.118	0.000***
	Aerobic – Strength	0.043	1.000
Total body fat (g)	Aerobic – Control	−1529.172	0.056*
	Strength – Control	−763.815	0.655
	Aerobic – Strength	−765.358	0.257
Total Lean Mass (g)	Aerobic – Control	178.018	1.000
	Strength – Control	−337.554	1.000
	Aerobic – Strength	515.571	0.401
Total body mass (g)	Aerobic – Control	−1330.408	0.298
	Strength – Control	−1100.479	0.463
	Aerobic – Strength	−229.929	1.000
Total body fat (%)	Aerobic – Control	−1.421	0.027**
	Strength – Control	−0.566	0.817
	Aerobic – Strength	−0.855	0.063*

*p < 0.1; **p < 0.05; ***p < 0.01.

A decrease in weight was seen in both the AT group and the ST group, with the loss being significant compared with the CG (AT-CG = −2.892 kg; ST-CG = −2.986 kg). When we compared both training programs, however, we found that weight loss was greater in the ST group, but the difference was minimal and not significant (AT-ST = 0.093 kg). BMI was also reduced in both the AT and the ST groups, and this decrease was significant compared with the CG (AT-CG = −1.075 kg/m^2^; ST-CG = −1.118 kg/m^2^). Comparison of both training programs showed that BMI was lower in the ST group, but the difference did not reach significance (AT-ST = 0.043 kg/m^2^).

An improvement in total body fat was observed in both the AT and the ST groups, but the only significant improvement occurred in the AT group compared to the CG (AT-CG = −1529.172 g). It should be noted that there was a greater loss of total body fat in the AT group compared to the ST group (AT-ST = −765.358 g).

No significant differences were found in total lean mass. Nonetheless, an increase in lean mass was seen in the AT group compared to the ST group and the CG (AT-ST = 515.571 g; AT-CG = −178.018 g), whereas the ST group had a decrease in total lean mass compared to the CG and the AT group (ST-CG = −337.554 g; ST-AT = −515.571 g). Total body mass decreased in both the AT and the ST groups compared to the CG but with no significant improvements (AT-CG = −1330.408 g; ST-CG = −1100.479 g). Furthermore, it should be noted that the AT group had a greater loss compared with the ST group (AT-ST = −229.929 g). Finally, total body fat percentage decreased in both the AT and the ST groups compared with the CG (AT-CG = −1.421%; ST-CG = −0.566%). However, only the losses in the AT group were significant in comparison with both the CG and the ST group (AT-CG = −1.421%; AT-ST = −0.855%).

Table 5 lists the means of all the variables comprising the IPAQ-SF according to each group and both before and after training. In addition, the Wilcoxon test was performed to determine whether the differences were significant between the pretest and the posttest in each group. No significant differences were observed in the control group (p-value greater than 0.1 in all cases), but in the AT and ST groups significant differences were observed in each of the variables (p = 0.000), which means that after training, the results on the IPAQ-SF were statistically better. In more detail, we can observe that in the AT group the number of METs increased in greater proportion in moderate, vigorous and total physical activity compared to both the ST group and the CG. Concerning the variable walking, the difference compared to the ST group and the CG did not reach significance but was nevertheless greater in the AT group.

**Table 5:** Statistical Analysis: IPAQ-SF. Comparison of two related samples: Means (MET-mins/week) and Wilcoxon Test.

	Mean		Z	*p*
	Pretest	Posttest		
**Control Group**				
Walking	1016.4	1004.3	−0.14	0.888
Moderate	344	370.67	−1.254	0.21
Vigorous	557.3	578.67	−0.71	0.478
Total	1917.7	1953.6	−1.068	0.286
**Aerobic Group**				
Walking	940.3	1527.1	−3.88	0.000***
Moderate	433.5	1437.5	−5.425	0.000***
Vigorous	878	3638	−5.402	0.000***
Total	2251.8	6602.6	−5.511	0.000***
**Strength Group**				
Walking	990.9	1626.2	−5.047	0.000***
Moderate	487	987	−4.299	0.000***
Vigorous	1017.8	1932.6	−4.728	0.000***
Total	2495.7	4545.8	−5.644	0.000***

*p < 0.1; **p < 0.05; ***p < 0.01.

Regarding diet, a paired samples test was performed for comparison of the means to determine whether there were possible differences in the diet before and after the treatment. No empirical evidence of statistically significant differences was found between the diet before and after treatment in the CG (difference pre-post = 12.13, t = 1,175; p = 0.260), the AT group (difference pre-post = 8.68, t = 0.975; p = 0.336) or the ST group (difference pre-post = 13.17, t = 1.416; p = 0.163).

## Discussion

The American College of Sports Medicine states that the minimum recommended amount of exercise to achieve health benefits is 30 minutes of moderate-intensity physical activity or 20 minutes of vigorous physical activity three times per week. With regard to weight loss, it has yet to be fully clarified whether specific types and amounts of exercises are more appropriate for this purpose, as there are individual variations. However, in general, any physical activity increases the probability of success in this regard^66^. The International Association for the Study of Obesity affirms that this amount of exercise is probably insufficient to bring about improvements in body composition and recommends 60–90 minutes of moderate physical activity per day^67^. The duration of our training programs is 60–90 minutes, three sessions per week and with increasing intensity throughout the twelve weeks of training, in accordance with the previous recommendations.

Several studies that have used both AT and ST detected no significant improvements in physical performance or adaptations of body composition, such as the studies by Izquierdo *et al*. (2004) and Sanal *et al*. (2013) in which DXA was used to analyze the different anthropometric variables as in our study, but with the difference that our study obtained significant improvements in weight loss, BMI, total body fat percentage and total body fat^68,69^. The results of our study, however, are consistent with other research in which mixed training programs are implemented^70,71^ with two and three weekly sessions, respectively, resulting in statistically significant improvements in body composition, specifically in weight and fat mass loss and increased lean mass.

It should be mentioned that most of the studies of overweight or obese young adults implemented mixed programs^66,69^ or a single training program while also intervening in dietary habits^16,17,72^. These studies were not focused exclusively on aerobic or strength work, which is the key innovative feature of our research.

A significant increase in metabolic expenditure was observed in both the AT and ST groups, results consistent with those of Vilaca *et al*. (2011) and Branco *et al*. (2019). Both of these studies involved concurrent training programs and recorded an increase in metabolic expenditure following the programs. This increase, however, was smaller, rising by a mean of 1200 MET-mins/week^73,74^, than that of our study in which a mean increase of 3200 MET-mins/week was recorded (Table 5). In our study, the AT group had a higher metabolic expenditure than the ST group and the CG, results that are in line with research by Bakker *et al*. (2017), Scharhag-Rosenberger (2017), Ramírez-Vélez (2017) and Ostendorf (2019) in which it was detected that in AT programs, metabolic expenditure is higher than when ST or mixed training is implemented, especially in moderate physical activity^75–80^. Nonetheless, significant improvements were found in moderate, vigorous and total physical activity.

The main findings of this research were that both AT and ST produce improvements in body composition and increase metabolic expenditure compared to the CG. In our study, based solely on administering AT or ST, improvements were observed in all the body composition parameters evaluated through DXA, which were a decrease in weight, BMI, total body fat, total body fat percentage, and total body mass as well as an increase in lean mass (Table 3), noting significant improvements in weight, BMI, total body fat and body fat percentage. The improvements in BMI and weight were greater in the ST group compared to the AT group and the CG. However, total body fat, total lean mass, total body mass and percentage of total body fat decreased more in the AT group compared to the other groups, and therefore the improvements in these variables were greater (Table 4).

Overall, the results in the AT group were better than those in the ST group since, despite this group obtaining greater improvements in two areas of body composition, the AT group showed improvements in all the anthropometric variables. Consequently, the results our study may contribute to improve the health and quality of life of young adults who are overweight or obese and offer them a possible solution to the problem affecting 38% of the current population, i.e., overweight. Potential associated health problems, such as cardiovascular or respiratory problems, can also indirectly be avoided without influencing diet.

## Conclusions

The data obtained in this study suggest that the 12-week training programs in both AT and ST were well tolerated, as the dropout rate was minimal, and generated significant improvements in the anthropometric variables of body weight, BMI, total body fat and total body fat percentage, highlighting that the ST group had a greater improvement in BMI and weight, while the AT group had better results in total body fat, total lean mass, total body mass and total body fat percentage. Thus it can be indicated that AT enabled better overall results to be obtained than ST both in comparison with the CG and between the two training groups.

Both training groups achieved an increase in metabolic expenditure, although the AT group showed greater increases compared to the ST group and the CG. These results suggest that the health and quality of life of overweight or obese individuals could be improved in a short period of time with exercise therapy alone. Future research should apply different physical exercise prescription models combining the two methodologies used in this research, such as high intensity interval exercises (HIIT), in order to examine their possible effects in this type of population and compare results.
